# Impact of a Pharmacist-driven Methicillin-resistant Staphylococcus aureus Polymerase Chain Reaction Nasal Swab Protocol on the De-escalation of Empiric Vancomycin in Patients with Pneumonia in a Rural Healthcare Setting

**DOI:** 10.7759/cureus.6378

**Published:** 2019-12-13

**Authors:** Precious Dadzie, Tyson Dietrich, John Ashurst

**Affiliations:** 1 Pharmacy, Kingman Regional Medical Center, Kingman, USA; 2 Pharmacy / Infectious Diseases, Kingman Regional Medical Center, Kingman, USA; 3 Emergency Medicine, Kingman Regional Medical Center, Kingman, USA

**Keywords:** mrsa, antibiotic stewardship, pneumonia

## Abstract

Introduction

Pneumonia caused by methicillin-resistant *Staphylococcus aureus* (MRSA) carries a high rate of morbidity and mortality. Many clinicians empirically treat those at risk of developing MRSA pneumonia with vancomycin. Several studies have identified a high negative predictive value of the MRSA polymerase chain reaction (PCR) nasal swab test in lower respiratory tract infections, suggesting it can be used to guide the de-escalation of empiric anti-MRSA therapy.

Objective

To evaluate the impact of a pharmacist-driven MRSA PCR nasal swab protocol on the de-escalation of empiric vancomycin in patients with pneumonia in a rural healthcare setting. Secondarily, to assess the rate of hospital length of stay, the rate of vancomycin-associated acute kidney injury, and in-hospital mortality after pharmacist-driven de-escalation of empiric vancomycin in patients with pneumonia.

Methods

A retrospective, single-center, pre-post cohort study was conducted in patients after the implementation of a pharmacist-driven protocol allowing pharmacists to obtain nasal swabs and PCR testing for MRSA in those on empiric vancomycin therapy for suspected MRSA pneumonia. Based on negative test results, pharmacists recommended a de-escalation of empiric vancomycin to the physician. Patients were included if they were adults at least 18 years of age, had a physician diagnosis of suspected or confirmed pneumonia, and initiated on at least one dose of intravenous vancomycin within 48 hours of admission.

Results

A total of 79 patients were identified for inclusion in the pre-protocol group (n=32) or post-protocol group (n= 47). The mean duration of vancomycin therapy in the pre-protocol group was 3.1 days as compared to 1.7 days in the post-protocol group for a 1.4 days reduction (p=0.044). There was no significant impact on the number of vancomycin cases de-escalated within 24 hours (p=0.14) but there was a significant reduction at 48 hours (p=0.01). Protocol implementation was associated with a reduction in the average length of hospitalization (8 versus 5.20 days, p=0.006). Neither group had a vancomycin-associated acute kidney injury or in-hospital mortality.

Conclusion

Among patients with suspected MRSA pneumonia, a pharmacist-driven MRSA PCR nasal swab protocol resulted in a significant reduction of empiric vancomycin duration of therapy without an adverse impact on clinical outcomes in a rural healthcare setting.

## Introduction

Pneumonia has been credited as one of the leading causes of infectious disease-related deaths in the United States [1]. *Staphylococcus aureus* was the second most common pathogen noted in all health-care-associated infections in the United States between 2011 and 2014 [2]. Although not a common pathogen seen in community-acquired pneumonia, methicillin-resistant *Staphylococcus aureus* (MRSA) is an emerging pathogen that accounts for between 20% and 40% of all nosocomial infections [3-4]. Current guidelines from the American Thoracic Society and Infectious Diseases Society of America (IDSA) recommend empiric treatment for those at risk of developing MRSA pneumonia despite its low prevalence in the community setting [5-6].

MRSA is a common pathogen that has been known to colonize the nares of patients and assay-confirmed infection has been shown to be predictive for the development of future MRSA infections [7]. The MRSA polymerase chain reaction (PCR) assay is a readily available test that carries a negative predictive value (NPV) of between 95$ and 99% for the detection of MRSA [8-9]. Data have shown that in the absence of nares, MRSA has been a negative predictor for the development of MRSA pneumonia [10]. The objective of this study was to evaluate the impact of a pharmacist-driven MRSA PCR nasal swab protocol on the de-escalation of empiric vancomycin in patients with pneumonia in a rural healthcare setting. The secondary objective was to assess the rate of hospital length of stay, rate of vancomycin-associated acute kidney injury, and in-hospital mortality after pharmacist-driven de-escalation of empiric vancomycin in patients with pneumonia.

## Materials and methods

Study design and patient selection

This retrospective, single-center, pre-post cohort study was conducted at a 235-bed hospital in rural Arizona, with an average annual emergency department volume of 50,000 patients. The pre-protocol group consisted of patients admitted from June 1, 2018, through September 30, 2018, and the post-protocol group consisted of patients admitted between November 1, 2018, and January 31, 2019. Adult patients at least 18 years of age with a physician diagnosis of pneumonia within 48 hours of admission and initiated on at least one dose of intravenous (IV) vancomycin for empiric MRSA coverage were included in the study. All patients included in the study had the diagnosis of pneumonia and were excluded if they were pregnant, admitted to the ICU, or received IV vancomycin for a concomitant infection. Additional exclusion criteria included if the nasal swab test was performed more than one month prior to the respiratory culture for patients admitted from the outpatient department, more than seven days prior to the respiratory culture for hospital-acquired cases, and if the nasal swab test was performed more than three days after the respiratory culture was collected. Vancomycin-associated nephrotoxicity was defined as a rise in serum creatinine of 0.5 mg/dl or 50% above baseline on at least two consecutive measurements after 72 hours of vancomycin and with no other apparent cause.

Protocol implementation

An MRSA PCR nasal swab protocol was developed as part of an antimicrobial stewardship initiative, and education regarding the protocol and clinical utility of the MRSA PCR nasal swab test was provided to all affected healthcare providers during October 2018. Hospitalists were educated individually and nurses received education via email communication. Education was provided to the pharmacist in multiple settings, including department meetings, individual interactions, and via email communication. The protocol was implemented on November 1, 2018, and authorized pharmacists to order an MRSA PCR nasal swab test for patients with pneumonia started on IV vancomycin. If the results of the swab were negative, the pharmacist would contact the provider to discuss the discontinuation of vancomycin. The ultimate decision to de-escalate vancomycin remained with the physician. Pharmacists and hospitalists received re-education throughout the study.

Statistical analysis

A sample size of 24 patients in each group was required to detect a one-day reduction in the duration of vancomycin therapy between groups, assuming an average baseline of 4+1.5 days. Categorical data were analyzed using the chi-squared test and continuous data were analyzed using the t-test. All tests were two-tailed, with statistical significance set at a p-value of less than 0.05.

## Results

In total, 888 patient records were reviewed and 79 patients were included in the final analysis. Overall, the baseline demographic data and clinical characteristics were similar in the pre- and post-protocol groups (Table 1).

**Table 1:** Patient demographics

Characteristics	Pre-protocol (n=32)	Post-protocol (n=47)
Median age (year)	69 (28-81)	71 (30-90)
Male	16 (50%)	32 (68.1%)
Congestive Heart Failure	7 (21.9%)	12 (25.5%)
Diabetes Mellitus, Type 2	12 (37.5%)	20 (42.6%)
Chronic Obstructive Pulmonary Disease	11 (34.4%)	19 (40.4%)
Immunosuppression	1 (3.1%)	0
Nursing Home/Extended Care Facility	6 (18.8%)	5 (10.6%)
Recent Hospitalization	12 (37.5%)	17 (36.2%)

There was no statistically significant difference in the number of vancomycin de-escalated patients within 24 hours between the pre- and post-groups (34% vs 51%; p=0.14). However, there was a significant difference in the amount of vancomycin de-escalation at 48 hours between the two groups (63% vs 87%; p=0.01). The duration of empiric vancomycin therapy was reduced by 1.4 days per patient in those with negative MRSA PCR swabs (3.1 days vs 1.7 days; p=0.04). Length of stay was also reduced by 2.8 days following the implementation of the protocol (p=0.006). There was no difference in the number of vancomycin-associated acute kidney injury or in-hospital mortality.

## Discussion

The overuse of anti-MRSA antibiotics has been associated with increased antibiotic resistance, nephrotoxicity, and increased healthcare costs and should be de-escalated once negative culture results are obtained [11]. Much like previous research, a pharmacy-driven protocol utilizing an MRSA nasal swab PCR in those suspected of having MRSA pneumonia resulted in a shorter duration of vancomycin therapy without increasing adverse events [11-12]. One could postulate that by decreasing the number of days a patient is on vancomycin for MRSA coverage in pneumonia that total healthcare costs would also be decreased because of fewer days in the hospital and less serum vancomycin levels being obtained. According to Smith et al., a $108 reduction in cost per patient was noted in medication-related costs following the implementation of a similar protocol [13].

Although a statistically significant number of vancomycin de-escalations was noted at 48 hours following a negative MRSA nasal PCR, a large number of cases could have been de-escalated sooner. This data coincides with previous data that protocol compliance has notably been poor for a pharmacy-driven protocol utilizing an MRSA nasal swab PCR to de-escalate vancomycin in pneumonia [11]. One would have imagined a larger number of de-escalations at 24 hours due to the Hawthorne effect but this was not seen till 48 hours. The exact mechanism for these findings is unclear but could be related to the ability to overcome previous practice habits [14]. 

The single-center retrospective nature of this study with a lack of randomization decreases external validity. This study had a relatively small sample size; however, this did not have a significant impact on outcomes. Another limitation was pharmacy staff compliance with protocol requirements was not evaluated. Despite evaluation, there were occasions when the MRSA PCR nasal swab test was not ordered, with a STAT priority resulting in a delay of the test result, which delayed potential recommendation for de-escalation.

## Conclusions

The implementation of a pharmacist-driven MRSA PCR nasal swab protocol had a significant impact on empiric vancomycin utilization in patients with pneumonia. No difference was seen in the rate of vancomycin-associated acute kidney injury or in-hospital mortality after pharmacist-driven de-escalation of empiric vancomycin in patients with pneumonia. Future studies should be aimed at continuing to validate the use of MRSA PCR nasal swabs for the de-escalation of empiric MRSA coverage in patients diagnosed with pneumonia. Missing in the already growing body evidence for this practice are multicentered, randomized controlled trials comparing MRSA PCR nasal swabs to traditional standards of practice.
